# The UK Eastern Network for Kidney Inflammatory Disease (ENKID) MDT at 24 months: advancing access to high-cost drugs, clinical trials, and complex case management in renal autoimmune diseases

**DOI:** 10.1186/s12882-026-04849-6

**Published:** 2026-03-28

**Authors:** Lucy Francis, Olivia Kanka, Ondrej Suchanek, Lisa C. Willcocks, Kevin W. Loudon, Clare Morlidge, Barbara Thompson, David C. Thomas, July Da Silva Araujo, Bernadette Laforteza, Praveen Jeevaratnam, Sapna Trivedi, Frances C. Hall, David R. W. Jayne, Rona M. Smith, Rachel B. Jones

**Affiliations:** 1 Department of Medicine, University of Cambridge, Cambridge, UK; 2 Addenbrooke’s Vasculitis and Lupus Department, Cambridge University Hospitals NHS Foundation Trust, Hills Road, Cambridge, CB2 0QQ UK; 3 East and North Hertfordshire NHS Teaching Trust, Stevenage, UK

**Keywords:** Glomerulonephritis, Regional multidisciplinary team meetings, Nephrology services, High-cost drugs, Autoimmune renal disease, Rare disease

## Abstract

**Background:**

Glomerulonephritis (GN) accounts for 20%–25% of the causes of chronic and end-stage kidney disease. The evolving treatment landscape with new targeted immunosuppressants has highlighted the unmet need for equity of access to specialist GN services. Regional networks and multidisciplinary team meetings (MDTs) provide access to clinical expertise, high-cost drugs (HCDs) and clinical trials, facilitating collaborative decision-making and optimal use of therapeutics to improve patient outcomes. Launched in May 2023 in the East of England (EoE), the Eastern Network Kidney Inflammatory Disease MDT (ENKID) provides a regional MDT framework designed to improve access to specialist expertise and HCDs- areas identified as lacking for 59% of nephrologists in a 2024 UK GN service survey.

**Methods:**

ENKID is led by the specialist vasculitis, lupus and primary GN service in Cambridge. A database build allows secure standardised data collection including referral indications and treatment decisions during fortnightly virtual regional MDTs.

**Results:**

Over 24 months, 229 discussions were conducted for 198 patients in 43 ENKID MDTs, averaging 15 attendees from 6 centres per meeting. Patients were referred for both clinical complexity and HCD use in 46% (*n* = 106), solely HCD access in 42% (*n* = 96) and for clinical complexity alone in 12% (*n* = 27). Clinical trial eligibility was an additional reason in 9% (*n* = 20). Diagnoses discussed were ANCA-associated vasculitis (33%, *n* = 65), membranous nephropathy (18%, *n* = 35), IgA nephropathy (19%, *n* = 38), lupus nephritis (15%, *n* = 29), minimal change disease/focal segmental glomerulosclerosis (7%, *n* = 14), rarer diseases (5%, *n* = 10) and diagnostic uncertainty (3%, *n* = 7). Including rituximab, a HCD was endorsed in 71% and alternative treatment recommended in 29% of referrals for HCD discussions. A wide geographical distribution of referrals was observed and facilitated widespread access to HCDs.

**Conclusions:**

The ENKID regional MDT addresses the increasing demand for specialist advice for patients with complex and rare kidney diseases. High attendance and case load underscore the need for this service, aligning with the UK government’s mandate for rare autoimmune disease. Integration of this MDT within the EoE NHS England renal network structure, providing governance, education and HCD access, serves as an exemplar for improving GN patient care and accessibility of UK services.

**Clinical trial number:**

Not applicable.

## Background

Globally, the burden of kidney disease is increasing, with the number of patients expected to reach 7.6 million in the UK by 2033 [1]. Glomerulonephritis (GN) accounts for 20–25% of chronic kidney disease and remains a major cause of progression to end-stage kidney disease [2]. Recent advances in understanding of immunopathogenesis, together with changes to the regulations of clinical trial endpoints, are leading to the approval of high-cost drugs (HCDs) for these rare diseases. The most recent examples of UK HCDs include avacopan for ANCA-associated vasculitis (AAV), voclosporin and belimumab for lupus nephritis (LN) and targeted-release budesonide and sparsentan for IgA nephropathy (IgAN) [3–7]. The therapeutic landscape continues to rapidly evolve; with multiple immunosuppressive agents currently in late-stage trials including pegcetacoplan for C3 glomerulopathy (C3G) [8], iptacopan for C3G [9], and obinutuzumab for LN [10].

There is a progressive shift towards early use of disease-modifying treatment aimed at preserving glomerular function and preventing irreversible damage. To accommodate this growing treatment potential, there is an increased need for specialist-led management of glomerulonephritis with equity in access to expertise and treatment, greater collaborative decision-making and experience sharing as more HCDs become available. The England Rare Diseases Action Plan (2021) identified addressing this need across rare diseases as a key national priority.

Indeed, the UK Rare Disease Framework and Getting It Right First Time Renal Medicine Report (2021) both flagged significant inequities in rare disease care [11, 12]. More recently, the 2024 Vasculitis Outcomes in Relation to Care Experiences (VOICES) study, which focused on patient experience in vasculitis, revealed major system-level challenges, including delays in diagnosis, difficulty accessing specialist input, and fragmented care. This study found that access to multidisciplinary team (MDT) care was associated with fewer emergency hospitalisations and serious infections than those without access to MDT care among 1,420 patients [13].

Regional MDTs aim to improve diagnostic accuracy, support timely treatment decisions in complex cases, and enable earlier intervention before irreversible kidney damage occurs. MDTs hope to reduce regional variation in access to HCDs, provide a platform to access specialist input for non-tertiary centres and promote care closer to home, where appropriate. For clinicians, it is an opportunity for education and helps maintain up-to-date knowledge of emerging treatments. Decisions made within an MDT are more likely to align with national guidelines compared to those made by individual clinicians working independently [14]. Additionally, MDTs support research participation; aligning closely with NHS priorities for rare disease care and networked service delivery.

There are eight renal networks across England [15]. Within this structure, the East of England (EoE) spans 19,119 km² and, in 2022, had a population of 7,082,983, served by 19 dialysis centres, 6 satellite sites, 6 integrated care systems, 662 general practices, and 148 primary care networks [16].

In this region, the Eastern Network for Rare Autoimmune Diseases (ENRAD) was established in 2016 to improve care for rare rheumatic autoimmune diseases. Its innovative MDT model received the British Society for Rheumatology’s Best Practice Award in 2018 [17]. The UK Rare Autoimmune Rheumatic Disease Alliance (RAIRDA) also highlighted ENRAD as a model for regional collaboration [18]. RAIRDA proposed ENRAD as a blueprint for similar networks, a point referenced in the very recent House of Commons Library briefing on rare autoimmune diseases [19]. Twenty-eight Rare Disease Collaborative Networks (RDCNs) exist as part of NHS England’s support for patients with rare disease. However these 28 disease groups do not include renal autoimmune diseases such as glomerulonephritis, lupus nephritis and renal vasculitis in their portfolio [20]. In view of the rising need for specialist glomerulonephritis care, the Eastern Network for Kidney Inflammatory Disease (ENKID) was established in May 2023 to parallel the existing rheumatic autoimmune regional network ENRAD and to integrate with the EoE renal network structure, providing a region-wide model bringing together expertise, specifically focused on autoimmune kidney disease. The ENKID initiative received funding and operational support from the EoE Renal Network and an industry partner.

## Methods

### ENKID MDT setup

**Fig. 1:**
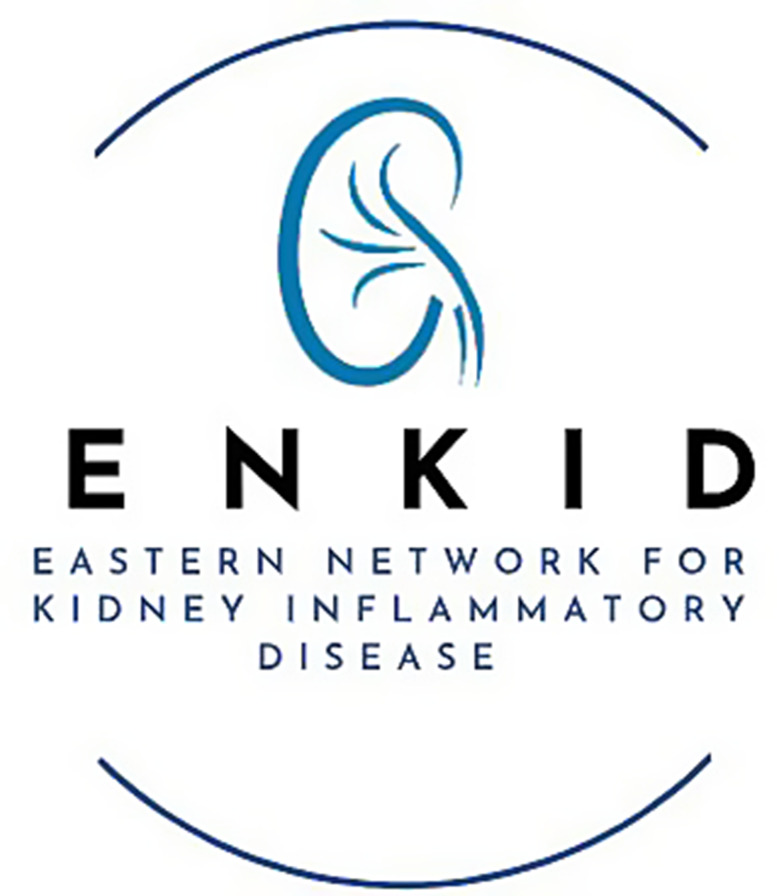
The Eastern Network for Kidney Inflammatory Disease (ENKID) logo

The vasculitis, lupus, and primary glomerulonephritis (GN) service at Cambridge University Hospitals (CUH), includes seven specialist consultants and four specialist nurses, providing consultant-led inpatient, outpatient, and urgent on-call services. As the largest service in the region, Cambridge is the host centre for ENKID. Other trusts in the region, including East and North Hertfordshire, also deliver inpatient, outpatient, and day-case services for patients with autoimmune kidney diseases. The ENKID MDT was developed using the existing service structure to formalise regional clinical advice for complex patients and recommendations on HCD use. Bespoke structured data-entry tools have been developed within the CUH EPIC electronic medical record system, using SmartForms and custom templates. This provides a standardised secure platform for patient data capture as well as reporting to track MDT actions and patients outcomes. A secure dedicated email address was set up for referrals which provides an audit trail. A service logo was also developed (Fig. 1). ENKID does not replace on-call urgent specialist advice but rather aligns with it.

The ENKID MDT launched in May 2023 and occurs twice monthly online via Microsoft Teams. The meeting accepts referrals related to primary GN, lupus nephritis and renal vasculitis and other complex multisystem diseases. Referrals to ENKID are initiated via a structured two-page proforma completed by the referring clinician. This captures key clinical information, including a summary of the patient’s presentation, past medical history, current and prior immunosuppressive therapy, relevant immunology and laboratory results, urinalysis (haematuria and proteinuria), imaging, histopathology and disease-specific metrics—such as the MEST score for IgAN, the National Institutes of Health activity and chronicity indices and the International Society of Nephrology/Renal Pathology Society (ISN/RPS) classification for lupus nephritis, and the Berden histological classification for AAV. Permission is obtained from the patient for discussion at a regional MDT. A specific ENKID MDT EPIC electronic medical record encounter is then created to capture referral data as stated in the referral proforma.

### MDT objectives

The main ENKID MDT objective is sharing expertise and collaborative decision-making in the management of patients with complex autoimmune kidney diseases. The key functions include providing recommendations and facilitating timely approval for high-cost drug use where MDT agreement is mandated, as well as supporting the implementation of national and local guidelines, utilising medical, pharmacist and nursing expertise. The MDT supports safe and efficient prescribing and supply of immunosuppressive therapies, improves access to clinical trials, and fosters networking among clinicians involved in renal autoimmune care. It also serves as a platform for education and upskilling, helping to build confidence and capacity in diagnosing and managing complex cases. ENKID reflects the growing need for dedicated, kidney-focused multidisciplinary care, bringing nephrologists and rheumatologists together in the management of complex autoimmune conditions with renal involvement, utilising organ-specific expertise needed to improve outcomes for patients with glomerular disease.

### MDT structure

MDT structures for renal autoimmune disease are not clearly defined, and the organisation of clinical services may vary considerably between regions. Recognising that not all cases are complex and that treatment decisions may be straightforward in some instances, we have set out a tiered structure for the EoE, comprising local, sub-regional (involving more than one hospital), and regional MDTs.

For rare renal autoimmune diseases with clinical complexity or highly specialised treatment pathways, where a regional MDT would be beneficial, specialist knowledge and multidisciplinary input from both medical and allied healthcare professionals are essential for effective functioning.

For ENKID in the EoE, we define the minimum requirements as four consultants, two specialist nurses, one specialist pharmacist, and dedicated coordination support. Specialist nurses contribute to governance processes subsequent to an MDT, such supporting data capture and managing HCD Blueteq forms. They also coordinate infusions and clinic referrals if this is required at CUH. The pharmacist oversees local implementation of HCD pathways, including formulary approval and dispensing, and advises on commissioning pathways (e.g. through integrated care boards) to support equitable access regionally. The pharmacist helps ensure national guidelines from the National Institute for Health and Care Excellence (NICE) are adhered to and their specialist expertise in immunosuppressive drug dosing and interactions, medication safety, prescribing and supply, and implementation planning are also vital to the MDT. An MDT coordinator is required for regional MDTs such as ENKID to provide administrative support by tracking referrals, liaising with referring centres, overseeing the ENKID referral mailbox, and scheduling meetings.

As a regional MDT, ENKID does not replace urgent telephone advice from a specialist centre, the transfer of unwell patients to tertiary care, or face-to-face outpatient review for patients with complex multisystem involvement.

### Data capture

A centralised database at CUH records MDT attendance, outcome decisions and reasons for recommendation or non-approval of HCDs. Completion of a Blueteq form is mandatory to obtain a Blueteq number, which is required for NHS England or ICB reimbursement of drug costs. A linked summary of the MDT discussion and letter is also sent to referring centres. This rigorous standardised documentation enables audit of decision consistency and equity of access. Automated outcome data reports can be extracted from the EPIC electronic health record system, either in aggregate or stratified by specific HCDs for repeated audit reviews.

## Results

### Referral and meeting attendance patterns

**Fig. 2:**
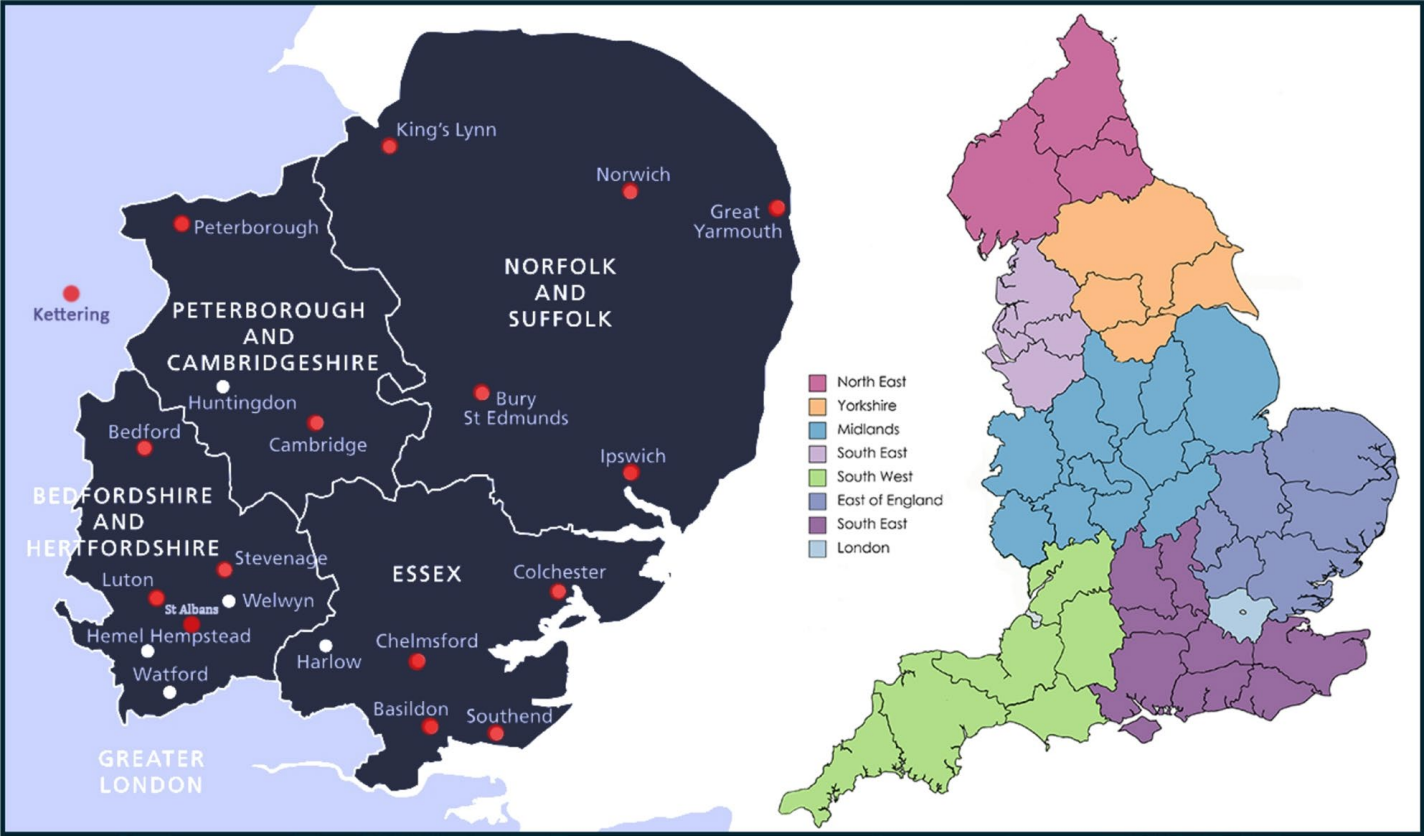
Geographical distribution of centres attending ENKID MDTs in 2023–2025. The left panel shows the location of renal centres from the East of England region that participated in ENKID MDT meetings (indicated by red dots), adapted from a Health Education East of England map [21]. Two outlying centres (Jersey and Gloucestershire) are not shown. The right panel illustrates the regional renal networks across England, with the East of England renal network highlighted in purple (created using www.mapchart.net)

**Fig. 3:**
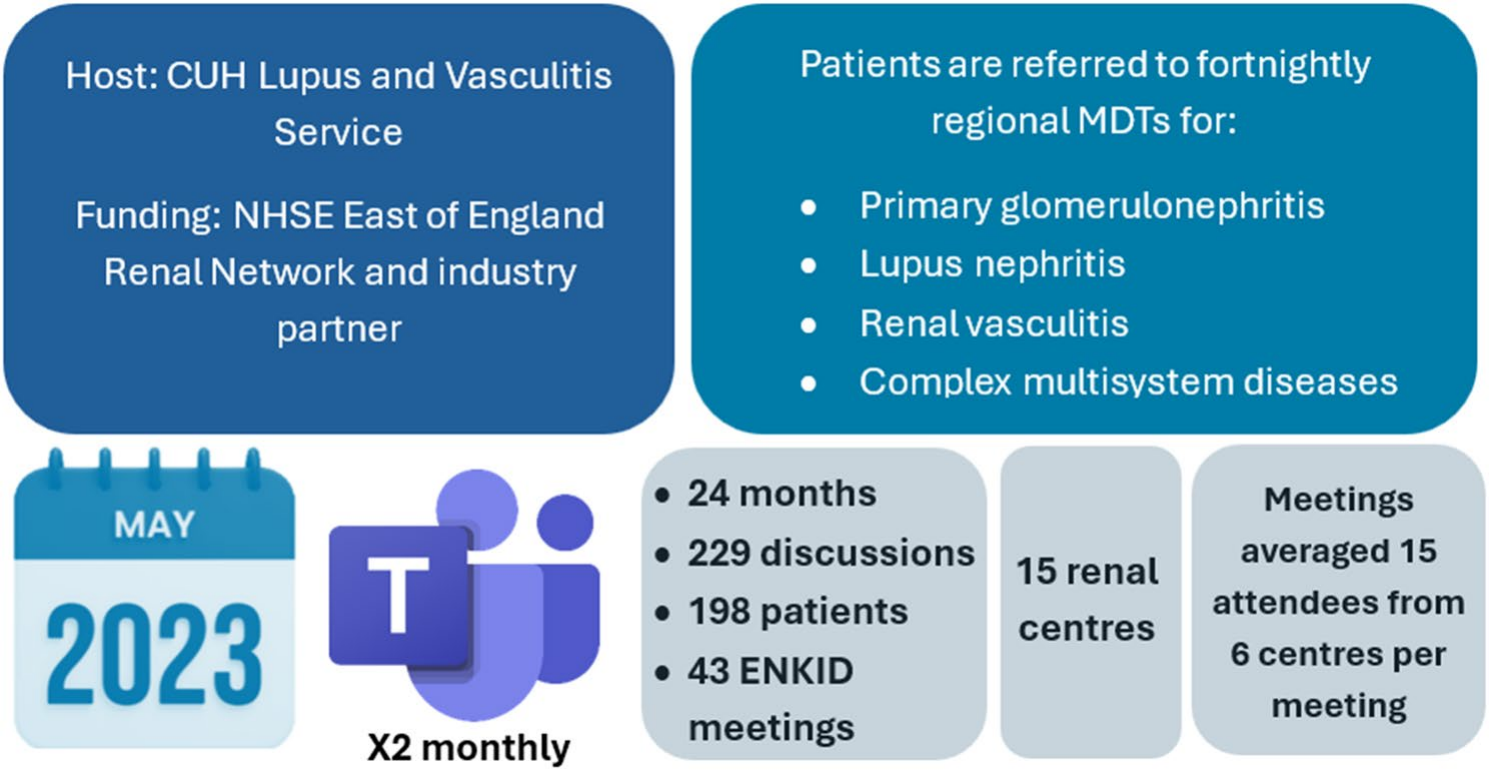
Key ENKID MDTs figures at 24 months

Rapid uptake and engagement with the ENKID MDT across the EoE was observed in 15 renal centres (Fig. 2). Over 24 months, 229 discussions were conducted for 198 patients in 43 ENKID meetings (Fig. 3). All referrals for discussion were made by consultants.

**Fig. 4:**
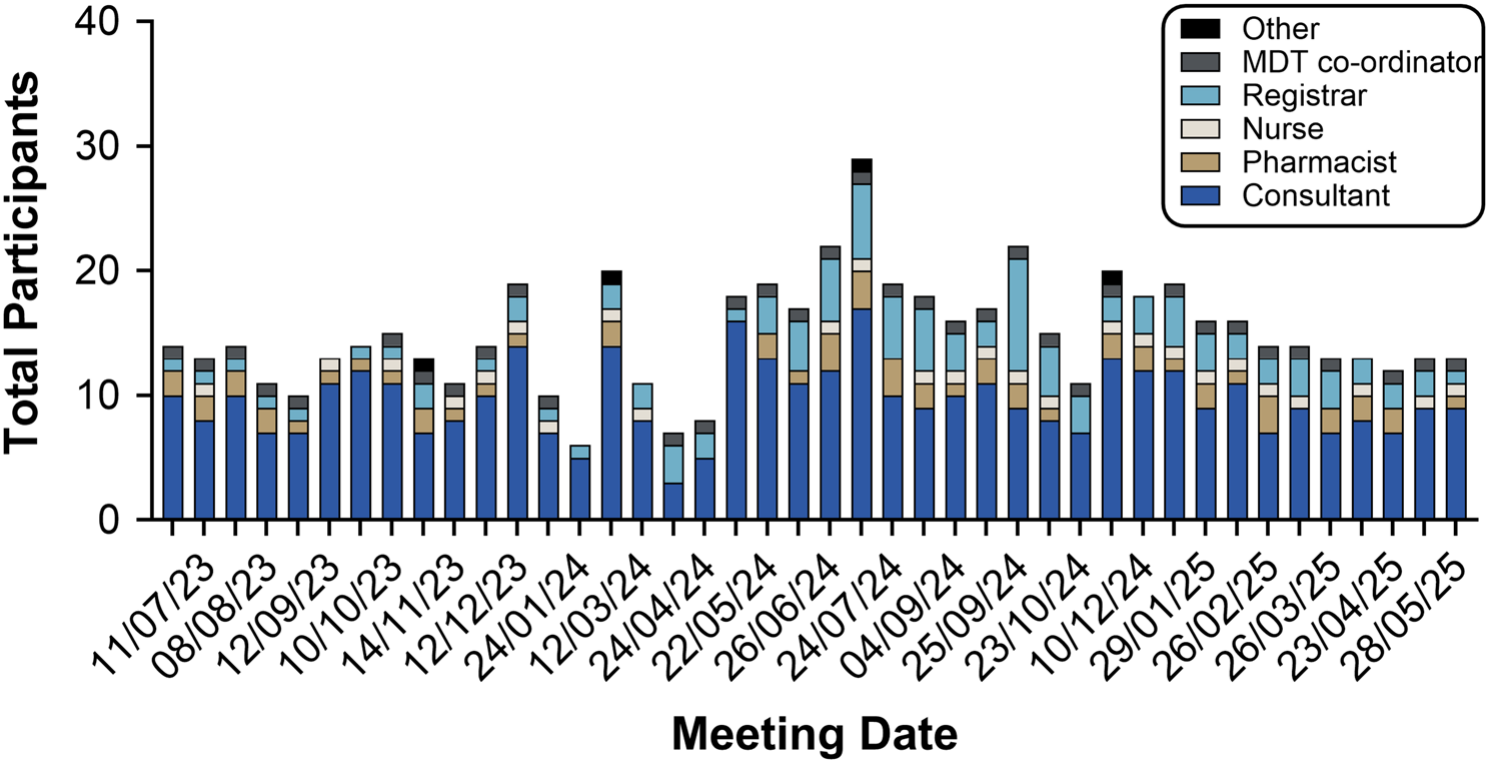
Attendance at ENKID MDT meetings between June 2023-May 2025. Stacked bar chart illustrating attendance by profession (consultants, registrars, specialist pharmacists, nurses, MDT co-ordinator and others) across individual MDT meetings

**Fig. 5:**
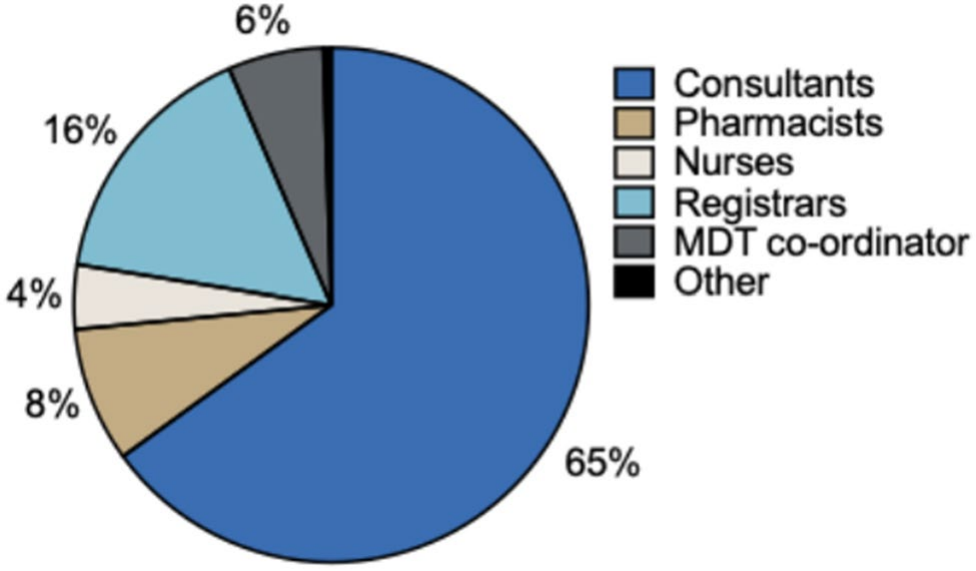
Typical composition of an ENKID MDT meeting by role. Pie chart illustrating the relative proportion of attendees by professional role, including consultants, registrars, specialist pharmacists, nurses, MDT coordinator, and others

Since 2023, each ENKID meeting has been attended on average by 15 professionals (Fig. 4) from six centres, with consultants comprising 65% of participants (Fig. 5). Up to three renal specialist pharmacists typically attend, including the HCDs and medicines optimisation pharmacist for the EoE Regional Renal Network. Their involvement ensures alignment with NHS commissioning policies and local pathways, supports HCD approval processes, and allows the MDT to address supply chain challenges. Specialist nurses (usually 1–2 per meeting) provide additional support with mandatory HCD paperwork, such as Blueteq forms if individual centres do not have access. We observed a steady increase in renal trainee attendance (Fig. 4). A dedicated MDT co-ordinator enables the effective organisation and delivery of meetings.

ENKID patients have been referred to discuss clinical complexity and HCD use in 46% (*n* = 106), solely HCD access in 42% (*n* = 96), and solely clinical complexity in 12% (*n* = 27). Additionally, in 9% (*n* = 20) clinical trial eligibility was an additional criterion for referral.

### Demographics and clinical characteristics of patients referred

**Fig. 6:**
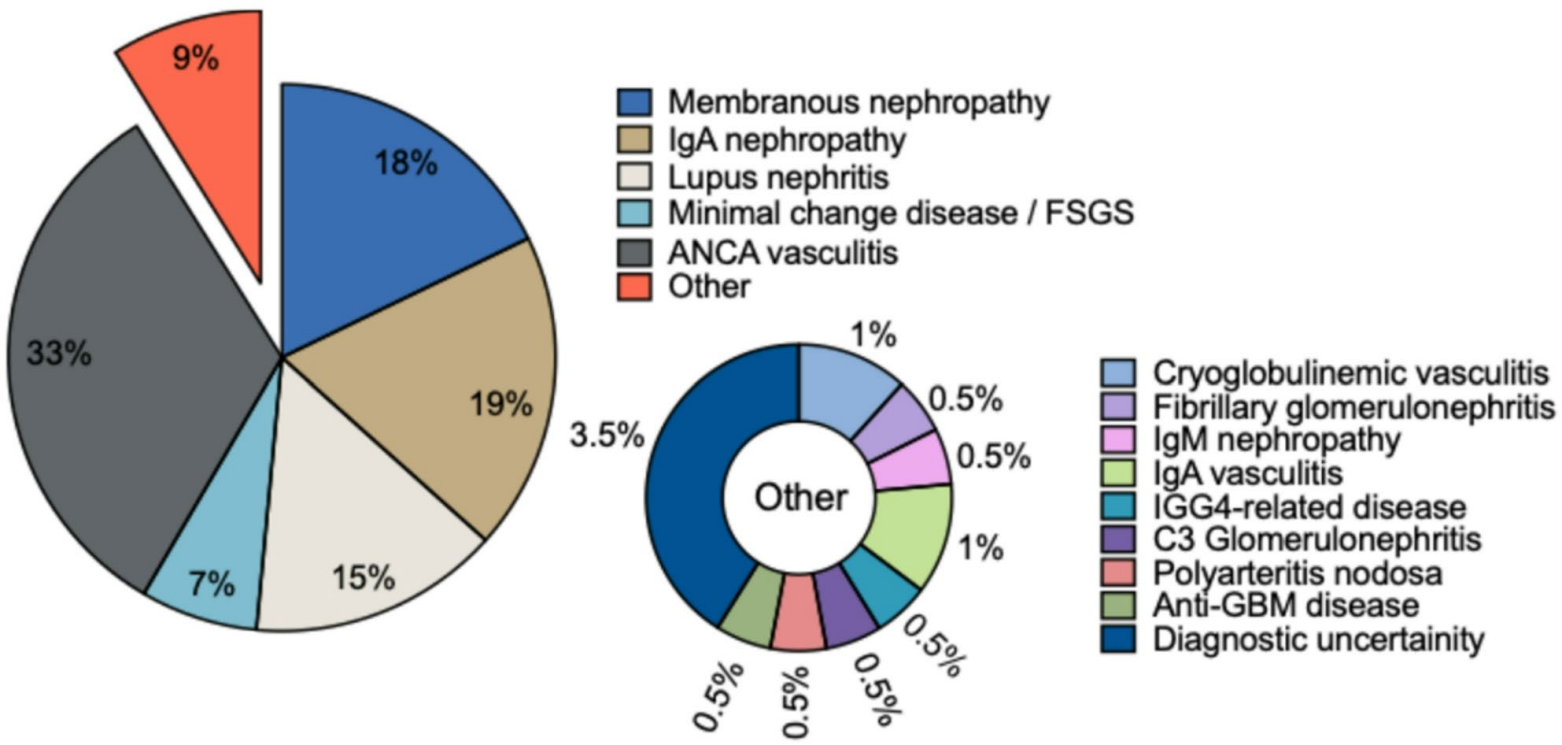
Distribution of renal autoimmune disease referrals to the ENKID MDT. Pie chart showing the distribution of 198 ENKID patient referrals by primary renal autoimmune disease. The orange “Other” category, representing rarer diagnoses, is further subdivided in the adjacent donut chart to illustrate the individual disease categories

Most referrals are for patients with ANCA vasculitis with renal involvement, followed by membranous nephropathy and IgA nephropathy (Fig. 6).

**Fig. 7:**
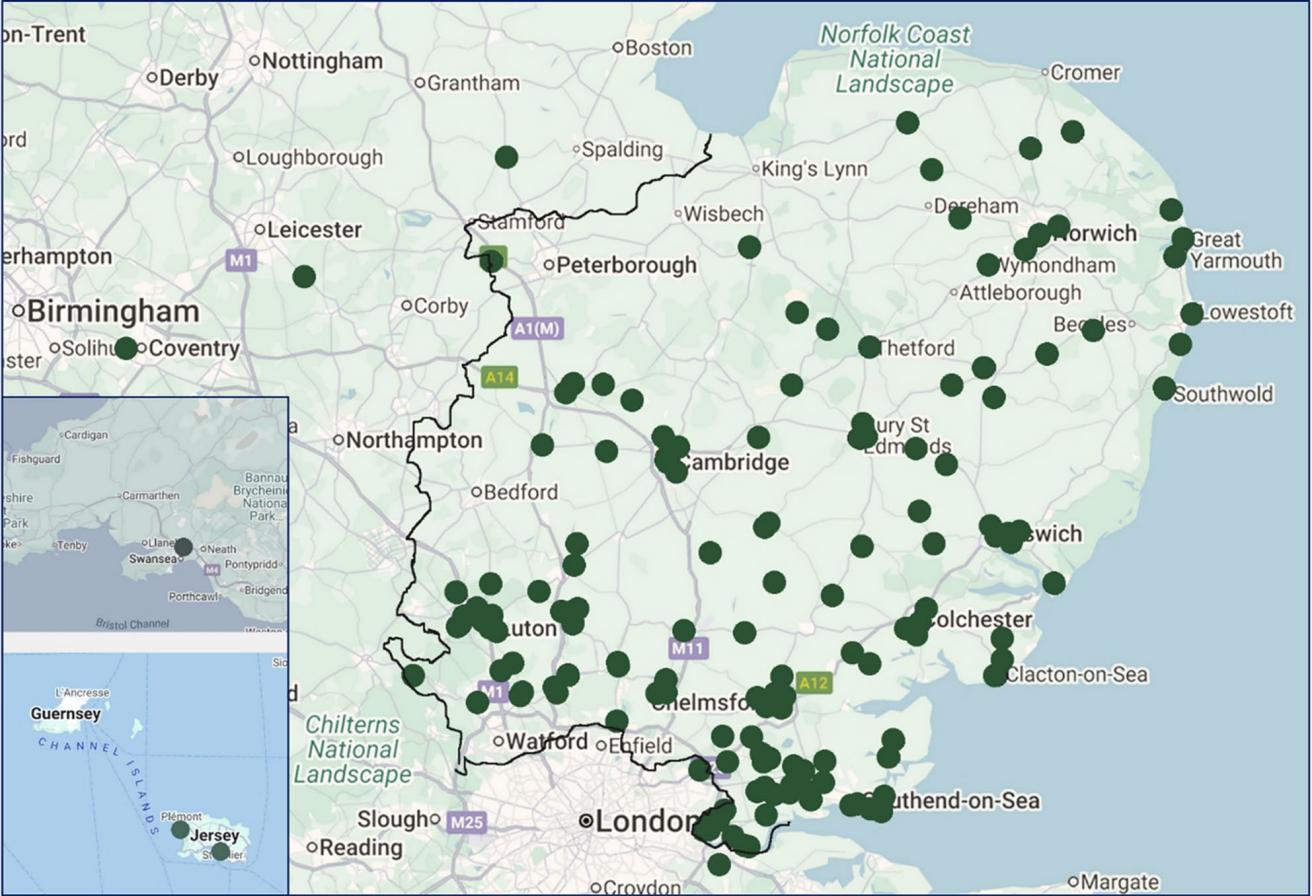
Mapping Equity of Access from GP Postcodes for ENKID Referrals between May 2023-May 2025. Figure adapted from Google Maps data using BatchGeo; boundary denotes the East of England region

Mapping the general practice(GP) postcodes of referred patients demonstrates a wide geographical spread across the East of England (Fig. 7). A small number of referrals originate from outside the region—including Wales, Coventry, and Jersey—typically due to patient relocation. This mapping exercise is beneficial in identifying potential gaps in service outreach and MDT coverage.

If use of a HCD has been approved in line with NICE or NHS policy, a Blueteq is issued either centrally through ENKID or locally by approved Trusts. If no Blueteq form exists, a separate letter will be sent to the trust pharmacist and the referring clinician, outlining the relevant policy, indication, and dosing details. A new HCD (avacopan, belimumab, voclosporin, targeted release budesonide, rituximab) was endorsed in 71% and alternative treatments recommended in 29% of referrals for HCD discussions. Treatment duration is reviewed according to NICE guidelines (e.g. avacopan at 1 year, targeted release budesonide at 9 months).

**Fig. 8:**
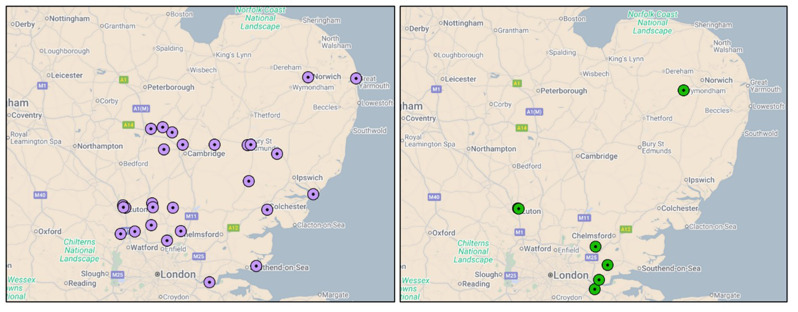
ENKID HCD recommendations for targeted release budesonide and voclosporin by GP location between May 2023-May 2025. Each dot represents a GP practice with a patient receiving a high-cost drug (HCD) recommended through the ENKID MDT. Some practices had multiple patients on the same drug, resulting in overlapping markers that may appear as a single point. Panels show distribution by drug: left (purple) – targeted-release budesonide (Mar 2024–May 2025; *n* = 28), and right (green) – voclosporin (*n* = 9). Targeted-release budesonide covers a shorter period as it became available later in the study timeframe

Equity of access to voclosporin, targeted-release budesonide, avacopan, and belimumab, based on ENKID recommendations, can be mapped across the region using GP postcode data alongside outcome capture (Fig. 8). Targeted-release budesonide and avacopan were the most frequently recommended therapies by the MDT, with a broadly even distribution across the region. This mapping approach enables the identification of potential gaps in access, which can be addressed through targeted outreach and educational initiatives.

**Fig. 9:**
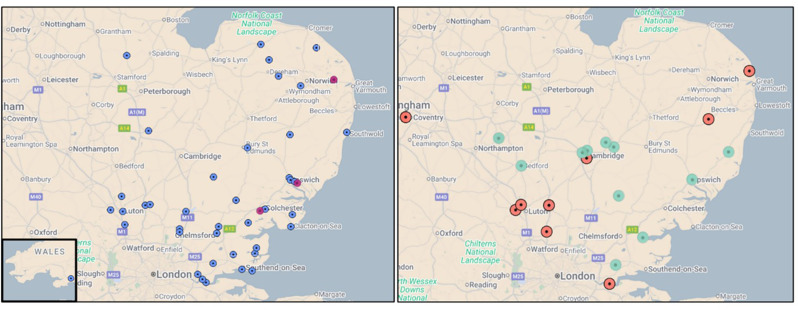
ENKID and ENRAD HCD recommendations for avacopan and belimumab by GP location between May 2023-May 2025. Each dot represents a GP practice with a patient receiving a high-cost drug (HCD) recommended through the ENKID or ENRAD MDT. Some practices had multiple patients on the same drug, resulting in overlapping markers that may appear as a single point. Panels show distribution by drug and network: left – avacopan via ENKID (blue, *n* = 42) and ENRAD (pink, *n* = 3); right – belimumab via ENKID (orange, *n* = 10) and ENRAD (green, *n* = 14)

Recommendations made via the ENRAD pathway also contribute to overall access for HCDs such as belimumab for lupus and avacopan for vasculitis (Fig. 9). Neither include local CUH Lupus and Vasculitis Service approvals.

With new renal autoimmune disease therapies expected over the coming years, equity of access will continue to be assessed using this mapping technique, as one means of monitoring regional distribution and access.

**Fig. 10:**
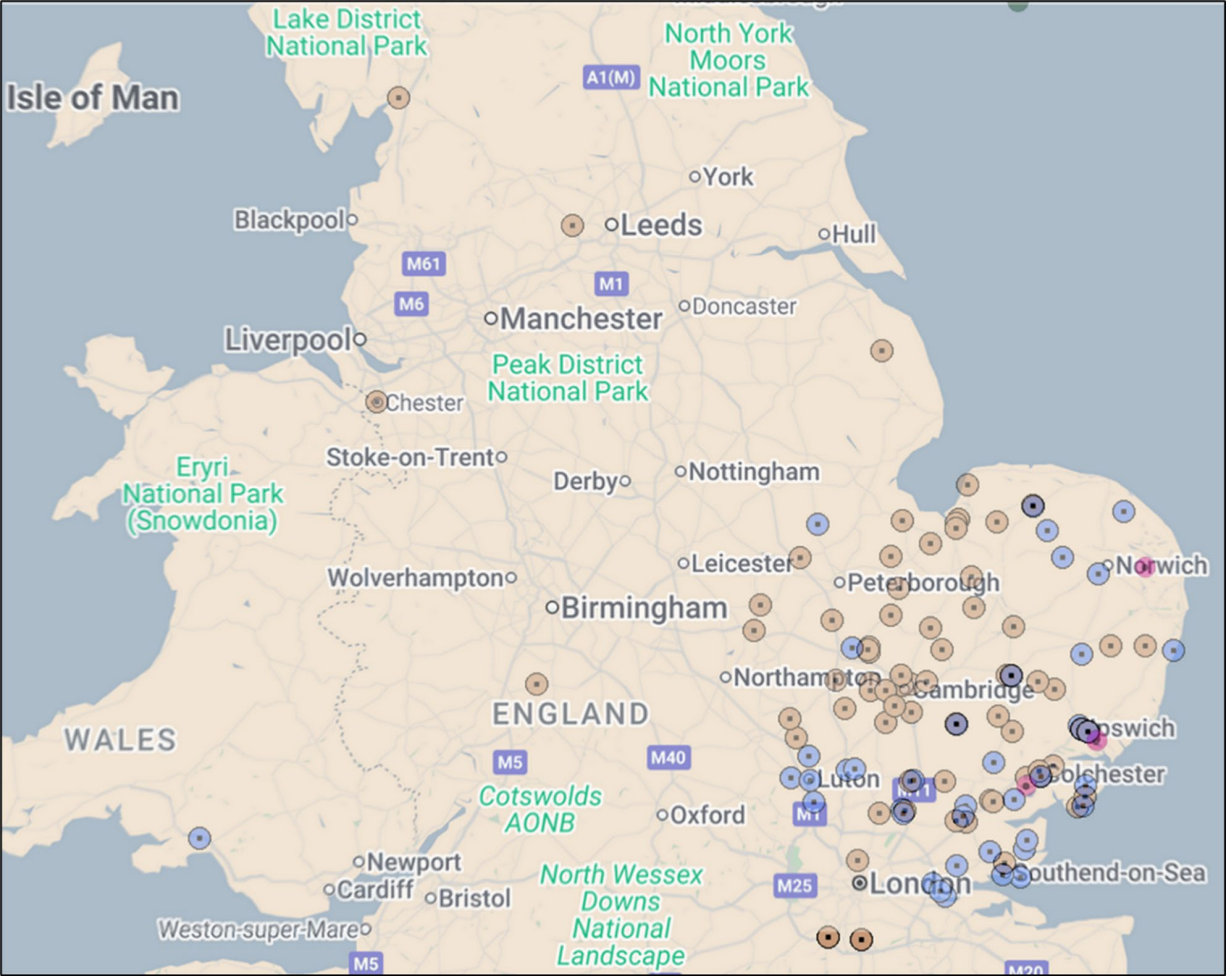
Avacopan recommendations by GP location across ENKID, ENRAD, and CUH Lupus and Vasculitis Service between May 2023–May 2025. Each dot represents a GP practice with a patient receiving avacopan. Purple dots show ENKID MDT renal recommendations (*n* = 42, including six referred to the CUH Lupus and Vasculitis Service); brown dots indicate local renal and non-renal Blueteq approvals for severe active AAV from the CUH Lupus and Vasculitis Service (*n* = 84); and pink dots represent recommendations via ENRAD (*n* = 3)

The CUH Lupus and Vasculitis Service cares for approximately 2000 patients; with 50% of referrals from the Cambridgeshire/Peterborough region and 50% of referrals from other centres across the country. Broad equity of access is demonstrated when mapping 84 avacopan recommendations from the CUH Lupus and Vasculitis Service local MDT decisions (in brown) alongside the 42 ENKID avacopan recommendations (in purple) (Fig. 10).

**Fig. 11:**
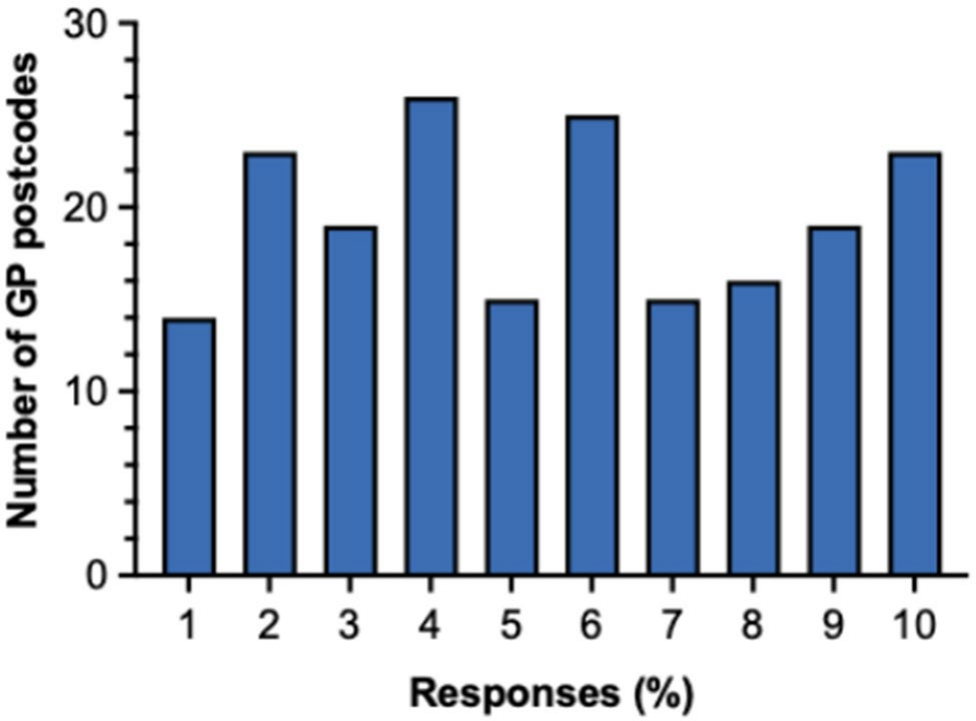
Distribution of GP postcodes for patients referred to ENKID between May 2023-May 2025, by 2019 English Index of Multiple Deprivation (IMD) decile. Bar chart showing, for each GP practice, a weighted average IMD score calculated from the relative deprivation of its registered patient population and converted into national deciles. The x-axis represents IMD deciles (1 = most deprived; 10 = least deprived), and the y-axis shows the number of ENKID referral GP postcodes (*n* = 195)

GP postcodes were linked to Lower-layer Super Output Areas (LSOAs) and matched to the 2019 English Index of Multiple Deprivation (IMD) deciles. IMD deciles represent the relative level of socioeconomic deprivation, calculated as a weighted average of the deprivation scores of each GP practice’s registered population [22]. IMD deciles were available for 195/198 records; three records with postcodes outside England (Jersey, Swansea) could not be assigned. The distribution spanned the deprivation gradient (Fig. 11): median IMD decile 6 (IQR 3–8). Overall, 19.0% (37/195) had GP postcodes in the most deprived deciles (1–2) and 21.5% (42/195) in the least deprived (9–10).

Beyond its clinical role, regional networks such as ENRAD and ENKID also play a central part in regional collaboration and knowledge exchange. Since 2024, ENRAD and ENKID have jointly hosted well-received regional educational days, including one focused on autoinflammatory/autoimmune disease and another on SLE and autoimmunity in pregnancy—promoting collaboration and multidisciplinary learning.

In parallel with these educational initiatives, efforts have been made to examine how ENKID is accessed and perceived across the network. For this purpose, an online survey was designed and disseminated throughout the ENKID network, to evaluate accessibility, utilisation and value in terms of specialist advice and access to HCDs.

**Fig. 12:**
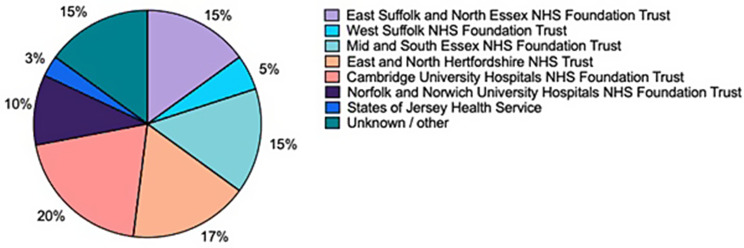
Distribution of survey respondents by NHS Trust and Jersey. Pie chart illustrating the proportion of survey respondents (*n* = 40) from each participating NHS Trust and Jersey

40 survey responses were received from more than ten EoE Renal Units, as shown in Fig. 12. Respondents included consultants (*n* = 29), registrars (*n* = 6), a consultant pharmacist (*n* = 1), pharmacists (*n* = 3), and other staff (*n* = 1). Of the 39 respondents, 21% attended all ENKID MDTs, 31% attended monthly, 41% attended only when discussing a patient, and 8% had not attended due to clinical workload.

**Fig. 13:**
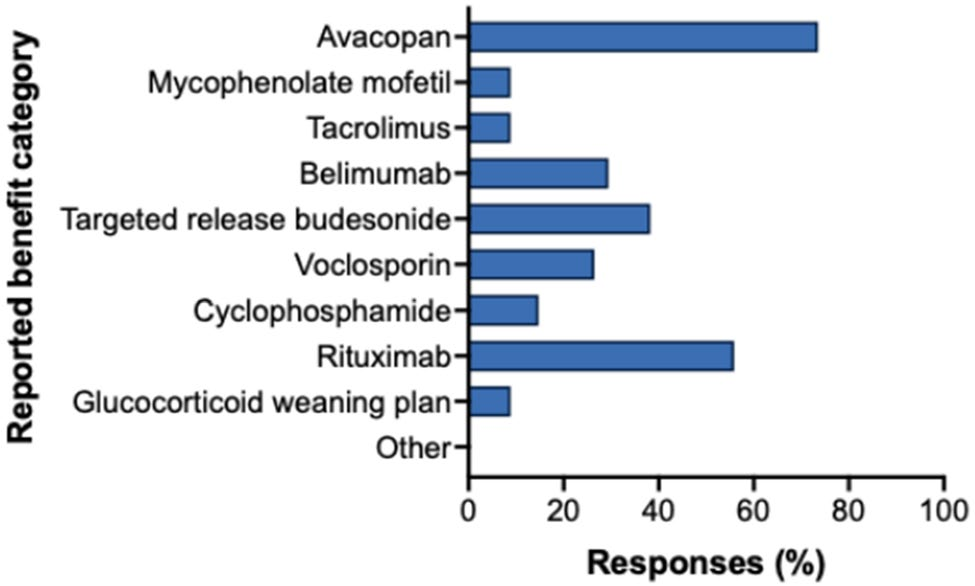
Treatment recommendations for cases discussed at ENKID meetings, as reported by survey respondents. Horizontal bar chart showing treatment recommendations as reported by survey respondents (*n* = 34). Multiple responses were permitted via drop-down selection

34 respondents reported receiving HCD approval for avacopan (74%), rituximab (56%), targeted-release budesonide (38%), belimumab (29%) and voclosporin (26%) (Fig. 13).

**Fig. 14:**
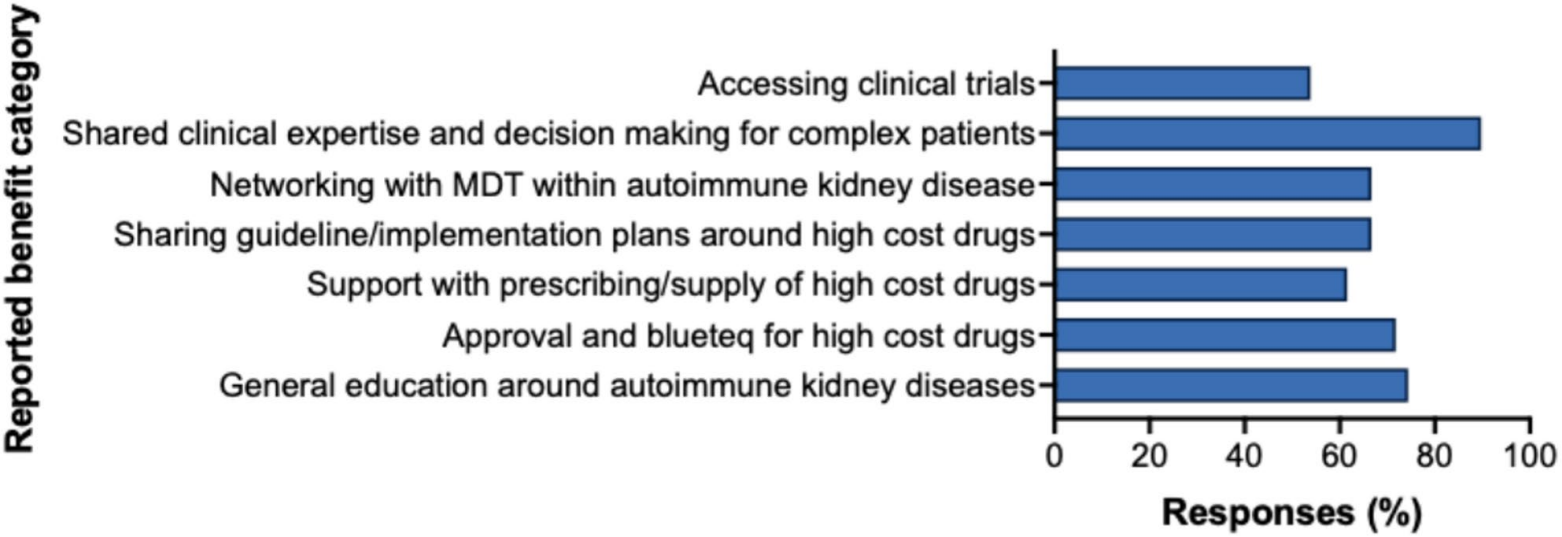
Reported benefits of ENKID MDT meetings. Horizontal bar chart showing the frequency of pre-defined benefits of ENKID MDT meetings as reported by survey respondents (*n* = 39). Multiple responses were permitted via drop-down selection

Reported benefits of ENKID were expert clinical advice for complex patients (90%), education on autoimmune diseases (74%), support for HCD access and Blueteq completion (72%), sharing approaches to HCD implementation/guidelines (67%) and clinical trial access (54%) (Fig. 14). Overall, 49% of respondents reported access to local MDTs for non-complex treatment decisions. Among these, 80% considered local MDTs sufficient for rituximab decisions in ANCA-associated vasculitis, LN and membranous nephropathy.

The sustained high case-load and attendance over 18 months demonstrates the ongoing need for ENKID. Ensuring optimal utilisation, accessibility, and value is now a priority. This survey was completed by 50% of ENKID attendees with representation from the majority of EoE renal units. MDT utilisation and value was high with 51% respondents attending meetings frequently irrespective of a requirement to discuss a case, and the majority reporting benefit across multiple domains including; clinical advice, education, HCD access and implementation.

## Discussion

The regional Eastern Network for Kidney Inflammatory Disease (ENKID) MDT model offers several benefits. For patients, it supports timely, collaborative decision-making aligned with national guidance, potentially leading to improved outcomes and reduced variation in care, with care closer to home. For clinicians, the MDT provides peer support and an opportunity to remain current in a rapidly evolving therapeutic landscape, addressing issues of professional isolation and variation in experience, particularly in smaller units. Access to a specialist forum also ensures greater consistency in therapeutic approaches. The educational value of these discussions, as highlighted in survey responses, contributes to long-term workforce development in nephrology and autoimmune care.

At a service level, ENKID meets the aims of the Rare Disease Action Plan by improving care coordination, enabling equitable access to specialist input, and fostering participation in research. The use of a centralised database facilitates outcome tracking, monitoring of equity of access to HCDs, and structured review of MDT decision-making. Importantly, ENKID is not a replacement for urgent referral or inpatient transfer of critically ill patients, but a complement to existing services by providing a proactive and structured forum for complex cases.

From an operational standpoint, key challenges during the development and early implementation of the MDT included funding for clinician time, database development, and coordinator support. Tertiary-centre consultants were contributing outside of contracted clinical time and with resource limitations, it was very challenging to establish an MDT coordinator. The service developed despite limited funding by assigning a dedicated research fellow to support database development and the timely and accurate communication of MDT outcomes. Data-sharing and governance challenges included ensuring appropriate patient consent, limited clinical detail within referral proformas, and the risk that ad hoc email-based case discussions were not consistently captured within the MDT framework. This was mitigated through enhanced administrative support, a dedicated ENKID service email address and standardisation of MDT proformas and processes.

The database structure continues to undergo iterative refinement to optimise data capture fields for audit and reporting purposes.

Ongoing audit enables centre participation to be tracked and the identification of centres who rarely attend the MDT is important. Reaching out to centres with initial infrequent attendance has subsequently improved participation.

Clear messaging during meetings has been important to ensure urgent clinical cases continue to be managed through on-call consultant to consultant phone advice, rather than awaiting ENKID MDT meetings. Positioning the MDT as an advisory forum that complemented local MDTs and prescribing processes, while aligning decision-making with NHS policy and NICE guidance, was critical to its acceptance. Transferable lessons for future MDTs, including the importance of early clarity around governance and scope, targeted outreach, dedicated funding and protected clinician time, and investment in administrative and data infrastructure. Looking ahead, with emerging positive phase 3 trial data in glomerular diseases and multiple therapies currently in late-stage development, the availability of high-cost drugs for renal autoimmune disease is set to grow substantially. This increase can be attributed to greater regulatory acceptance of surrogate renal endpoints such as proteinuria, alongside advances in understanding disease immunopathogenesis. The current therapeutic pipeline includes complement inhibitors [9, 23], B-cell–directed agents (including type II anti-CD20 and B-cell activating factor/APRIL pathway blockade) [10], and endothelin/renin–angiotensin–aldosterone system antagonists [7], with momentum further strengthened by recent approvals [3, 4, 24]. As multiple therapies enter clinical practice in parallel, specialist input will be essential to ensure their optimal and cost-effective use, including tailoring treatment to disease phenotype and evaluating potential combinations.

Multidisciplinary forums such as ENKID can facilitate shared learning about emerging therapies, support phenotype-based treatment decisions and enable the collection of real-world experience, potentially enabling the gradual refinement of therapeutic approaches. Mapping regional access to HCDs, as demonstrated, will also be key to maintaining consistency and ensuring equitable implementation.

Beyond renal autoimmune disease, the ENKID model for rare renal autoimmune conditions aligns with NHS England rare disease collaborative networks for knowledge sharing and care delivery. Glomerular diseases, including lupus and vasculitis, are complex multi-system disorders that frequently require input from rheumatology, respiratory, dermatology, and other specialties. Increasing the expertise of renal autoimmune MDTs across specialties may improve coordination between disciplines. This approach supports national priorities to reduce fragmentation of care, align treatment pathways across multiple specialties, and ensure that patients benefit from innovations as they emerge. Notwithstanding these benefits, the MDT model remains referral-dependent; patients are reviewed only if recognised and referred. Important upstream determinants such as timely recognition of renal autoimmune disease and rapid access to diagnostics (e.g., acute renal screens and renal biopsy) remain issues in rare renal autoimmune disease and lie largely outside the MDT’s remit, contributing to delays in diagnosis and treatment. The MDT could however offer a platform to strengthen regional education through increased involvement of registrars, internal medicine trainees, specialist nurses and pharmacists, broader participation from non-renal professionals, and targeted educational initiatives. Downstream constraints such as clinic and infusion capacity, and local commissioning decisions can also impede implementation of MDT recommendations, with the potential to widen inequities if unaddressed. The MDT can serve as a forum to identify and escalate such disparities. Ensuring sustainability will require protected time for MDT activity to be incorporated into consultant, nurse, and pharmacist job plans. This aligns with the 2025 British Society for Rheumatology service specification within the Management Recommendations for ANCA-Associated Vasculitis, which emphasises inclusion of MDT participation and dedicated time for prescribing, administration, and monitoring of high-cost drugs within clinical job planning [25].

Future work will focus on increasing patient involvement in ENKID training days, generating early real-world outcome data on high-cost drugs, undertaking pharmaceutical equity analyses, and exploring formal integration of renal autoimmune MDTs within NHS commissioning structures. The ENKID MDT model will be further developed to incorporate patient representative perspectives and allow evaluation of MDT processes. Patient representation will focus on reviewing MDT function and how outcomes are communicated to patients, contributing to improvements in transparency, patient information materials and the overall experience of MDT-informed care.

As a new regional renal autoimmune MDT in the UK, ENKID provides a potential blueprint for wider collaboration between centres. In future, it aims to engage with emerging regional MDTs to harmonise referral criteria, case review processes, and high-cost drug governance. Over time, this could provide a framework for a national case-discussion forum for rare or complex autoimmune kidney diseases, meeting periodically and operating under appropriate governance and information-sharing agreements. Linkage with national datasets such as NHS Digital will enable benchmarking of diagnostic patterns, high-cost drug use, and variation in care across regions. Generalisability nationally will be dependent on the availability of expertise, digital infrastructure, clinician time, administrative support, and geographic distribution of sites and services within regions. Globally the availability of emerging therapies, differing reimbursement mechanisms, and governance frameworks also underscores the need for context-specific adaptation.

There is a strong case for wider adoption of similar MDT models across the UK, consistent with recommendations from the UK Rare Autoimmune Rheumatic Disease Alliance and the NHS Rare Disease Framework [10, 17]. Formal evaluation of effectiveness, scalability, and sustainability will be key to supporting national adoption.

## Conclusions

This new regional MDT highlights the integral role of regional MDTs in improving care for patients with complex and rare kidney diseases. High attendance and case load underscore the unmet need for this specialist service, aligning with the UK government’s mandate for rare autoimmune disease. Integration of this novel network within the existing renal regional network structure in the EoE has helped provide a framework for multidisciplinary governance in recommendations for HCDs, a forum to discuss complexity, and provides training and patient outcome reporting.

## Data Availability

The data used in this paper is stored on EPIC with the Vasculitis/Lupus Department at Cambridge University Hospitals NHS Trust, UK. The datasets used and analysed during the current study are available from the corresponding author on reasonable request and ethical approval.

## Abbreviations

AAV: ANCA-associated vasculitis
CUH: Cambridge University Hospital
C3G: C3 glomerulopathy
EoE: East of England
ENKID: Eastern Network for Kidney Inflammatory Disease
ENRAD: Eastern Network for Rare Autoimmune Diseases
ESKD: End stage kidney disease
GP: General Practice
GN: Glomerulonephritis
HCDs: High-cost drugs
IgAN: IgA Nephropathy
IMD: Index of Multiple Deprivation
ISN: International Society of Nephrology
RPS: Renal Pathology Society
LN: Lupus Nephritis
NICE: National Institute for Health and Care Excellence
MDT: Multidisciplinary team meeting
RAIRDA: Rare Autoimmune Rheumatic Disease Alliance
RDCNs: Rare Disease Collaborative Networks
VOICES: Vasculitis Outcomes in Relation to Care Experiences
